# Implications for introgression: has selection for fast growth altered the size threshold for precocious male maturation in domesticated Atlantic salmon?

**DOI:** 10.1186/s12862-018-1294-y

**Published:** 2018-12-18

**Authors:** A. C. Harvey, O. T. Skilbrei, F. Besnier, M. F. Solberg, A.-G. E. Sørvik, K. A. Glover

**Affiliations:** 1 Institute of Marine Research, P. O. Box 1870, Nordnes, NO-5817 Bergen, Norway; 2 Department of Biology, University of Bergen, P. O. Box 7803, N-5020 Bergen, Norway

**Keywords:** Precocious males, Hybridisation, Fitness, Growth, Salmon

## Abstract

**Background:**

Mature male parr (MMP) represent an important alternative life-history strategy in Atlantic salmon populations. Previous studies indicate that the maturation size threshold for male parr varies among wild populations and is influenced by individual growth, environmental conditions, and genetics. More than ten generations of breeding have resulted in domesticated salmon displaying many genetic differences to wild salmon, including greatly increased growth rates. This may have resulted in domesticated fish with the potential to outgrow the size threshold for early maturation, or evolution of the size threshold of the trait itself. To investigate this, we performed a common-garden experiment under farming conditions using 4680 salmon from 39 families representing four wild, two wild-domesticated hybrid, and two domesticated strains.

**Results:**

Domesticated salmon outgrew wild salmon 2–5-fold, and hybrids displayed intermediate growth. Overall, the numbers of MMP varied greatly among families and strains: averaging 4–12% in domesticated, 18–25% in hybrid, and 43–74% in the wild populations. However, when the influence of growth was accounted for, by dividing fish into lower and upper size modes, no difference in the incidence of MMP was detected among domesticated and wild strains in either size mode. In the lower size mode, hybrids displayed significantly lower incidences of mature males than their wild parental strains. No consistent differences in the body size of MMP, connected to domestication, was detected.

**Conclusions:**

Our data demonstrate: 1- no evidence for the evolution of the size threshold for MMP in domesticated salmon, 2- the vastly lower incidence of MMP in domesticated strains under aquaculture conditions is primarily due to their genetically increased growth rate causing them to outgrow the size threshold for early maturation, 3- the incidence of MMP is likely to overlap among domesticated and wild salmon in the natural habitat where they typically display overlapping growth, although hybrid offspring may display lower incidences of mature male parr. These results have implications for wild salmon populations that are exposed to introgression from domesticated escapees.

**Electronic supplementary material:**

The online version of this article (10.1186/s12862-018-1294-y) contains supplementary material, which is available to authorized users.

## Background

Atlantic salmon (*Salmo salar* L.) display high levels of phenotypic and life history plasticity, both within and among populations and regions [1]. Some of this variation is underpinned by genetic variation, and may reflect adaptations to local environmental conditions [2, 3]. Such plasticity can also benefit populations by buffering against environmental change [4]. Although “land-locked” Atlantic salmon populations spending their entire life cycle in freshwater exist [5], the most common life-history strategy for this species involves anadromy [6]. Anadromous populations reproduce and develop in freshwater for 1–4 years before undergoing smoltification and then migrating to sea to grow [1]. After spending 1–3 years at sea, maturing adults return to their natal rivers to complete the life-cycle by spawning.

Within salmon populations, a further flexibility in life history occurs, involving a reproductive strategy whereby some males precociously mature as parr at considerably smaller sizes than anadromous males in freshwater [6]. Mature male parr (MMP) will wait near an anadromous couple and sneak in to deposit their sperm after the female releases her eggs [7]. In this way, MMP can successfully reproduce with anadromous females [8, 9], although their spawning success is highly variable [9–13]. While the incidence of MMP varies among populations throughout the salmons’ native range, they represent an important component as they increase the effective population size and contribute to population structure [9, 11]. The life-history strategy adopted by male parr is in part influenced by life-history trade-offs between early maturation (MMP) with limited reproductive contribution but increased probability to survive to the reproductive age, and late (adult) maturation and potentially greater reproductive contribution but low probability to survive to the reproductive age [14].

The male parr maturation strategy observed in Atlantic salmon is regarded as a threshold trait, as the fish need to reach a certain threshold value of an underlying continuous trait to adopt a specific reproduction tactic [15, 16]. In salmon, the underlying trait is growth rate or body size, and the threshold value is a body size that an individual should attain within a specific time to transition into a MMP [17]. Smoltification and migration do not occur at the same time as maturation, therefore, it has been proposed that there are two successive developmental thresholds in salmon: one for early male maturation and one for smoltification [18]. Salmon with high growth bypass the early maturation threshold and will develop into anadromous individuals after achieving the smoltification threshold, while those which grow slower will either achieve the maturation threshold and develop into MMP or continue to grow and reach the smoltification threshold at a later stage [18]. These alternate developmental pathways often manifest as a bimodal size distribution as parr segregate into the alternative phenotypes [18, 19]. Studies show that MMP, before becoming mature, can display higher growth rates compared to their non-maturing conspecifics [15, 20]. For any given individual male, the trade-off between parr maturation vs adult maturation is linked to individual growth opportunities, environmental conditions, genetics and possibly their interaction [21–23].

Domesticated escapees interbreeding with wild conspecifics represents one of the biggest challenges to the sustainability of the salmon aquaculture industry [24], and escapees represent a major threat to the genetic integrity and evolutionary trajectory of wild populations [25]. Although the reproductive success of escaped adult domesticated salmon is lower than wild salmon [26, 27], the reproductive success of domesticated MMP has been reported to be higher than that of wild mature parr under experimental conditions [28]. Together, these data suggest that if the offspring of escaped domesticated fish develop into MMP, this may “fast-track” further gene flow into wild populations. Indeed, models of introgression have indicated that if the offspring of domesticated escapees develop as MMP, this will have a significantly higher influence on introgression than if they do not [29].

It has been suggested that selection may shift the thresholds that trigger alternative life-histories [30]. Domesticated males that mature as parr are undesirable in the salmon farming industry due to negative effects on growth performance, and the industry often employs photoperiod and temperature manipulation techniques to ensure domesticated salmon develop rapidly and enter smoltification [3]. Domesticated salmon outgrow wild salmon several-fold under aquaculture conditions due to generations of selection for increased growth [31, 32], while in the wild, the observed growth differences between domesticated and wild salmon are much less [33, 34]. If the threshold size in domesticated salmon has been changed via domestication and directional selection for increased growth, this may quantitatively influence this trait in wild populations when introgression of domesticated escapees occurs.

Previous studies have reported that the size threshold for development of MMP differs between wild populations [14, 20], and that domesticated salmon, and their hybrids with wild salmon, display lower incidences of MMP [14, 35, 36]. However, these studies have been limited to comparisons either between several wild populations or between a low number of wild and domesticated strains. In the present study we investigated whether domestication and selection for higher growth has altered the propensity to mature as male parr or caused the evolution of its size-threshold value by investigating growth and incidence of MMP in 39 families of two domesticated, two wild-domesticated F1 hybrid and four wild strains reared in a common garden experiment design under farming conditions.

## Methods

### Family production

**Fig. 1:**
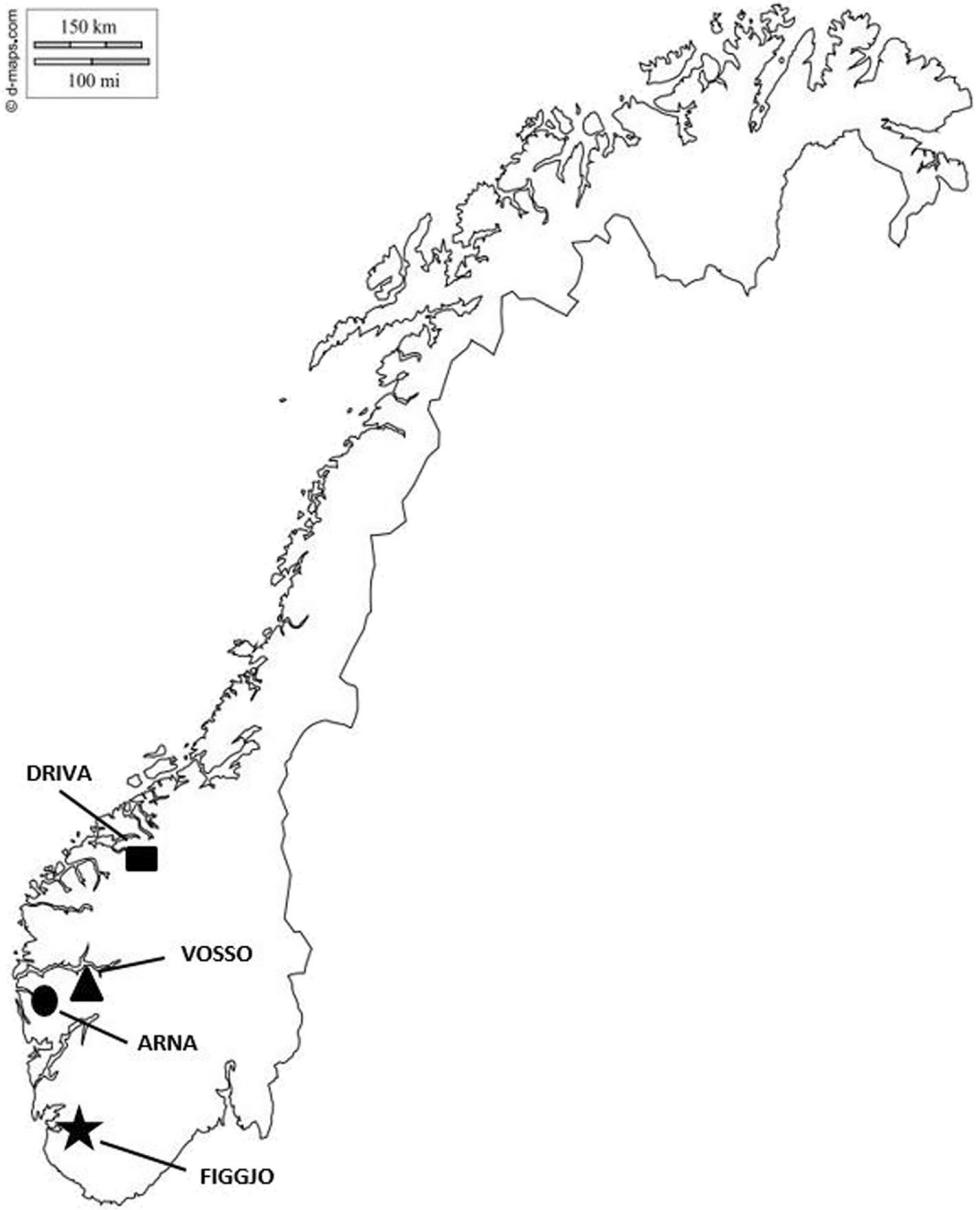
Map of Norway indicating the rivers of origin of the wild strains

Experimental families were created in November 2012 at the Matre Research Station, owned by the Institute of Marine Research (IMR), Norway. Adult brood fish were collected from four wild populations and two commercial domesticated strains to create eight experimental strains: four wild, two domesticated and two F1 wild-domesticated hybrid strains. Wild parental fish were either sampled directly from the river (Arna, Figgjo and Vosso, although in the latter brood fish were partially reared in the Norwegian Gene Bank until the smolt stage) or collected from the Norwegian Gene Bank for Wild Atlantic salmon (Driva) (Fig. 1). Brood fish were verified as originating from the wild based on scale readings [37]. The two commercial domesticated strains used were Mowi and Salmobreed. As a comparison between the two domestic strains was not the focus of this experiment, they were randomly anonymised as Domestic 1 and Domestic 2 and are referred to as the domesticated strains throughout. The backgrounds of each wild and domesticated strain used in the present experiment have previously been described in detail in [38].

A total of 39 families were created using five families per strain, apart from one wild strain (Driva) where only four families were included. Five dams were each crossed with one of five males within each strain, apart from Driva, where three females were crossed with one of two males (eggs from one female were divided and fertilised by both males). The Hybrid 1 families were produced by crossing Figgjo females with Domestic 1 males, and Hybrid 2 families were produced by crossing Domestic 2 females with Vosso males, therefore the eggs from these females were divided and fertilised by different males. The eight experimental groups are from here on referred to as experimental strains. Refer to Additional file 1: Table S1 for family design.

### Rearing conditions & sampling

Fertilised eggs were kept in the dark until the eyed-egg stage in single-family hatchery trays. In week 4 of 2013, 60 eyed eggs from each of the 39 families were mixed into two replicate tanks (2340 fish in each tank) prior to hatching. In week 14, prior to swim up, fish were transferred to two communal replicate tanks and the experiment was initiated. Experimental tanks were 1 m × 1 m × 30 cm with an average flow of 20 (±5) L/min. Photoperiod was maintained as LD (light to dark ratio) 12:12 and water temperature was ambient (average: 10 °C, range: 4.3–14.7 °C). Fish were fed constantly in the 12-h day period with commercial pellets from Skretting using automatic feeders, and using the producers feeding tables (Skretting AS, Norway). Pellet size was adjusted as the fish grew. Due to increasing biomass, the two replicates were each split into two tanks (total = 4), after which the tanks were split again (total = 8 replicate tanks). Rearing conditions were identical across all tank replicates and mortality was low (< 10% overall throughout the entire experiment).

The experiment ran from transferral to start-feeding tanks in April 2013 (week 14), until January 2014 (week 3) when fish were sampled (= 41 weeks). Upon termination, all fish were euthanised according to standard protocol with an overdose of Finquel®. Thereafter, they were sampled in two steps. The first step involved taking a random sample of fish from each tank (238–272 fish per tank) that were measured for wet-weight, fork length and fin clipped for parentage analysis (Table 1). In addition, these fish were dissected and sexed and classified as mature male parr (MMP), immature male parr (IMP) or female. The second step involved individually checking all the remaining un-sampled fish in the tanks for male parr maturation (27–61 of the remaining fish were found to be MMP per tank). Any MMP that were found in the second sampling step were thereafter sampled for biological measurements and fin clipped. This procedure provided both a random sample of fish from each family (to reconstruct family growth patterns), as well as ensuring that all mature males produced in the study were sampled (to gain as much information about the biology of the MMP as possible).

**Table 1:** Sex-specific and pooled growth data for each strain

	Females				Immature male parr				Mature male parr (MMP)				Pooled			
	n	W (g)	L (cm)	CF	n	W (g)	L (cm)	CF	n	W (g)	L (cm)	CF	n	W (g)	L (cm)	CF
		(± SE)				(± SE)				(± SE)				(± SE)		
Arna	132	37.31 (1.63)	14.40 (0.25)	1.10	69	39.96 (2.23)	14.79 (0.35)	1.11	51	15.59 (0.88)	11.05 (0.18)	1.10	252	33.44 (1.21)	13.82 (0.19)	1.10
Driva	85	18.61 (1.69)	11.42 (0.33)	1.01	40	12.38 (1.88)	9.90 (0.44)	1.00	51	10.65 (0.53)	9.99 (0.14)	1.03	176	14.89 (0.99)	10.66 (0.20)	1.01
Figgjo	132	14.14 (1.07)	10.35 (0.22)	1.04	72	13.79 (1.48)	10.13 (0.32)	1.04	73	11.90 (0.52)	10.16 (0.13)	1.09	277	13.46 (0.65)	10.24 (0.14)	1.05
Hybrid 1	144	35.17 (1.44)	14.16 (0.22)	1.10	93	36.34 (1.86)	14.39 (0.28)	1.09	32	22.34 (2.32)	12.16 (0.35)	1.13	269	34.05 (1.07)	14.00 (0.17)	1.10
Dom 1	131	66.76 (2.20)	17.76 (0.23)	1.12	122	69.30 (2.19)	18.08 (0.21)	1.12	18	22.33 (2.93)	12.13 (0.48)	1.15	271	64.95 (1.61)	17.53 (0.18)	1.12
Vosso	121	35.45 (1.60)	14.38 (0.25)	1.07	32	27.22 (4.16)	12.51 (0.70)	1.05	93	16.74 (1.04)	11.19 (0.19)	1.09	246	27.30 (1.17)	12.93 (0.19)	1.08
Hybrid 2	129	45.88 (2.04)	15.33 (0.28)	1.13	100	47.13 (1.89)	15.75 (0.26)	1.12	24	19.58 (1.67)	11.73 (0.30)	1.15	252	43.75 (1.38)	15.16 (0.19)	1.12
Dom 2	119	64.74 (1.72)	17.71 (0.18)	1.12	120	69.08 (1.55)	18.06 (0.14)	1.15	6	22.17 (3.80)	12.28 (0.68)	1.14	245	65.82 (1.22)	17.75 (0.12)	1.14

N; number of fish, W (g); average weight in grams, L (cm); average length in cm, CF; condition factor, SE; standard error. Pooled: all fish from the representative sampling (1988 in total). Dom; domestic

**Table 2:** Output of the final model for the LME investigating growth of MMP among strains

N	Response	Random	effects			Fixed	effects					
	Variable	Variable	Chi.sq	Chi.df	*P* value	Variable	Sum Sq	Mean Sq	Num Df	Den Df	F value	P value
654	Log Weight	Tank	5.77	1	0.0163	Strain	15.27	2.18	7	18.98	4.06	0.007
		Sire	7.25	1	0.0071							
		Dam	12.68	1	0.0004							

N; number of fish. Log weight; log10 (wet weight) at termination. Chi.sq.; the value of the Chi square statistics. Chi Df; the degrees of freedom for the test. P value; *P*-value of the likelihood ratio test for the random effect. Sum.Sq; sum of squares. Num Df, numerator degrees of freedom. Den Df; denominator degrees of freedom based on Sattherwaithe’s approximations. F; F-value

### DNA extraction & family assignment

DNA was isolated from the parental fish and the sampled offspring using a Qiagen DNeasy® 96 Blood & Tissue Kit in order to assign each individual back to family of origin. Six microsatellite loci were amplified in one PCR multiplex: SsaF43 [39], Ssa197 [40], SSsp3016 (GenBank # AY372820), MHCI [41], MCHII [42], and SsOSL85 (GenBank # Z48596.1). PCR products were resolved on an ABI3730XL sequencer and genotypes were manually scored using GeneMapper V5.0. Fish were assigned back to family using the Family Analysis Program (FAP) (v3.6) [43].

### Statistical analysis

Statistical analysis was carried out using R version 3.3.2 [44].

A linear mixed model (LMM) was fitted using the *lmer* function from the *lme4* package [45] to investigate whether the average body size for parr maturation had evolved in domesticated or hybrid salmon using all sampled mature male parr (*n* = 654). The response variable was the log-transformed and centred individual wet-weight at the end of the experiment. The full model included the fixed factor covariate of strain (8 levels). Sire, dam and tank replicate effects were included in the model as random intercept terms. The significance of the fixed and random effects were investigated using the *step* function from the *lmerTest* package, which performs backwards selection on both the fixed and the random effects to determine the most parsimonious model [46]. Post-hoc pair-wise comparisons between strains were performed using the *lsmeans* function in the *lsmeans* package by calculating the differences in least square means using the final model with a Tukey adjustment for multiple comparisons [47]. The final model fit was examined by diagnostic plots.

To compare the growth of MMP to the growth of immature males and females, a second growth analysis was conducted on the dataset of randomly sampled individuals (*n* = 1988). The response variable was wet-weight as above, and the full model included the fixed factor covariates of strain (8 levels) and sex/maturation status (3 levels, MMP, IMP and females) and their interaction term. Random effects were specified as above. The significance of the fixed and random effects were investigated as above. Where applicable, pair-wise comparisons between strains and between sexes (i.e. the interaction term) and for each fixed covariate were performed using the *lsmeans* function as above [47]. The final model fit was examined as above.

**Fig. 2:**
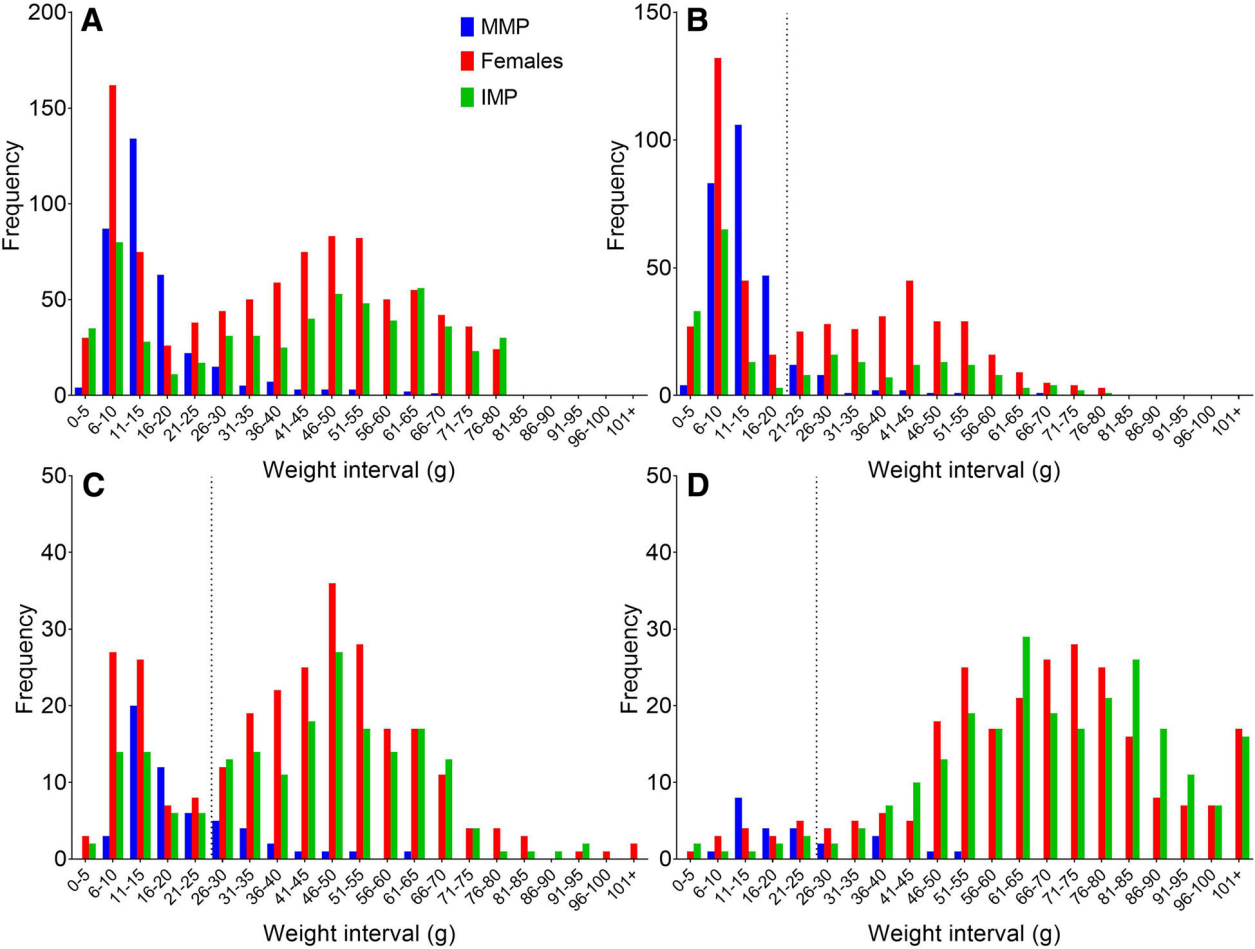
Weight frequency distributions for: all strains (**a**), wild (**b**), hybrid (**c**), and domestic strains (**d**). The vertical stippled line indicates the predicted average size threshold between the lower and upper mode for the wild (19 g), hybrid (26 g) and domesticated (26 g) strains in these data. Note scaling differences on Y-axis

There were large observable differences in the numbers of MMP among the strains, which appeared to be linked to size and whether an individual was located within lower or upper modes of the bimodal weight distribution of all the fish (Fig. 2). Therefore, the data was divided into a lower and upper size mode and each mode was analysed separately to investigate differences in the incidences of MMP among the strains. Fish were separated into lower and upper size modes using the Cassie method [48–50] based on their final weights and using 1) the wild fish size mode division for all strains, and 2) using the size divisions for each genetic group (i.e. wild, hybrid, and domesticated) separately. Analyses were conducted using both criteria to ensure that results were unbiased by any among-population differences in size distributions. A generalised linear mixed model (GLMM) was used to investigate whether strain or final weight affected the incidence of mature male parr in experimental fish in each of the size modes. The response variable, MMP, was binary, and thus the Bernouli distribution was used with the default logit link function. The full model included the fixed factor covariate of strain, the fixed continuous covariates of average logged and centred weight and the second-order polynomial of average logged and centred weight, and all logical two-way interaction terms. The GLMM model was fitted using the *glmer* function from the *lme4* package [45] and the fixed effect structure of the final model was determined by backward selection using the *drop1* function based on Akaike information criterion (AIC) values [51]. A low AIC indicates a good fit, and if model AIC’s differed by less than 2, the simplest model was chosen. Random effects were specified as above for growth. Where applicable, pair-wise comparisons between the strains were performed using the *lsmeans* function as above [47]. The final model fit was examined as above.

## Results

### The data

Of the 4680 fish included in the experiment, between 238 and 272 were randomly sampled from each of the 8 replicate tanks (2008 fish randomly sampled in total). After visual inspection of the gonads of the remaining un-sampled fish, a further 307 fish were determined as MMP, and sampled. Of the 2315 fish that were sampled in total, 18 could not be unambiguously assigned back to a single family using the selected set of microsatellites. In addition, three fish displayed abnormal condition factors, indicating data-recording errors. These 21 individuals were removed from further analyses, leaving 2294 sampled fish with complete family and phenotypic data. Therefore, the final data set for analysis consisted of a random sample of 1988 fish, of which 996 were male (mature and immature), and 992 were female (immature), in addition to an additional sample of 306 MMP. The first family-based growth analysis was computed on all MMP (total MMP = 654), while the second analysis was computed on the random sample of both sexes (*n* = 1988). The analysis of the incidence of MMP was conducted on the random sample of males only (*n* = 996). Average measurement data for each strain is given (Table 1).

### Growth

On average, domesticated strains were largest, hybrid strains were intermediate, and wild strains were smallest (Table 1, Fig. 2). In the model containing only the MMP individuals, strain was significantly associated with weight, and after correction for multiple comparisons among the strains, Driva was significantly smaller than Domestic 1, and Figgjo was significantly smaller than both Domestic 1 and Domestic 2 (Tables 2 and 3). There were no significant differences in the size of MMPs between any of the other strains.

**Table 3:** *P* values of the Tukey adjusted multiple comparisons of the weight of all MMPs among the strains. Significant p values are in bold

	Arna	Driva	Figgjo	Hybrid 1	Dom 1	Vosso	Hybrid 2	Dom 2
Arna	–							
Driva	0.84	–						
Figgjo	0.63	1.00	–					
Hybrid 1	0.98	0.43	0.11	–				
Dom 1	0.08	**0.01**	**0.00**	0.29	–			
Vosso	1.00	0.84	0.62	0.98	0.07	–		
Hybrid 2	1.00	0.81	0.59	1.00	0.12	1.00	–	
Dom 2	0.63	0.13	**0.04**	0.97	0.96	0.60	0.45	–

Using all randomly sampled individuals, the final weight model contained strain, sex/maturation status and the interaction between strain and sex/maturation status (Additional file 1: Table S2). Although there were observable differences in the mean size of MMP among the strains (Table 1), and the previous model containing only the MMP highlighted significant differences among some strains (above), in the model containing all randomly sampled individuals these differences were not found to be statistically significant after correction for multiple comparisons (Table 1, Additional file 1: Table S3A). After adjustment for multiple comparisons, immature males were significantly largest, females were intermediate and MMP were significantly smaller than both. The final weights of Domestic 1 and Domestic 2 were similar when compared at the equivalent sex or maturity level; similarly, the hybrid strains did not grow significantly different to each other in any sex or maturity level (Table 1, Additional file 1: Table S3B, S3C). Although the mean sizes of the hybrids were, for the most part, intermediate between their wild and domesticated parental strains, after correction for multiple comparisons there was no significant difference in growth between the hybrid strains and their domesticated parental strains in any sex or maturity level (Table 1, Additional file 1: Table S3). Immature hybrid individuals were significantly larger than their immature half-siblings in both hybrid crosses (Table 1, Additional file 1: Table S3).

### Incidence of mature male parr 

**Fig. 3:**
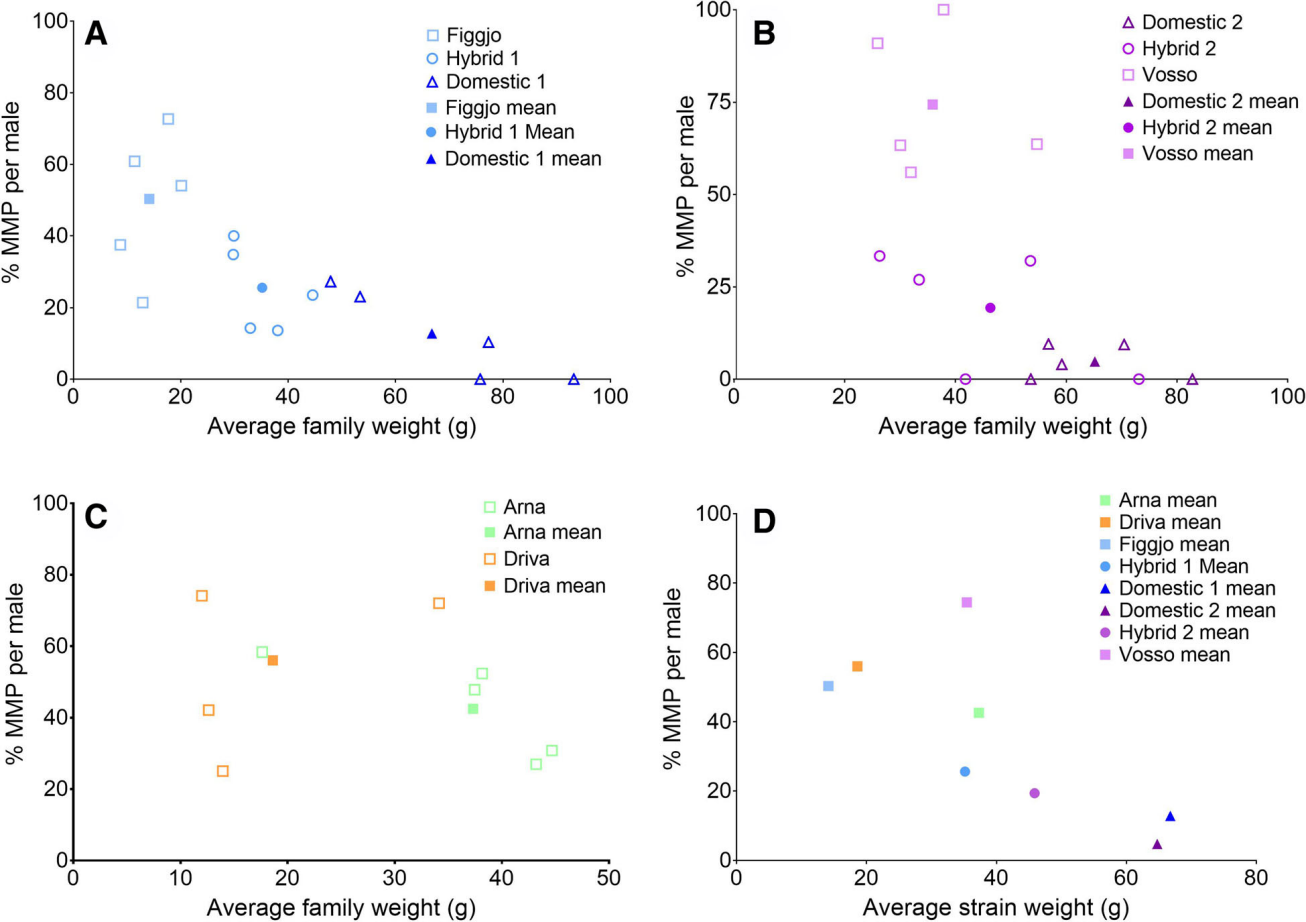
Percentage mature male parr (MMP) versus average weight per family (**a**-**c**) and per strain average (**d**). The open symbols represent family averages while the filled symbols represent strain averages. Strains are grouped in the figures per those that share half siblings in **a** (Figgjo strain, Hybrid 1 and Domestic 1) and **b** (Vosso strain, Hybrid 2 and Domestic 2), while the Arna and Driva strains are grouped to conserve space in **c**. Average family weight per strain for each of the three sex categories

Of the 996 randomly sampled males, 348 (35%) were mature. Overall, the incidence of MMP varied greatly among families and strains, with the highest incidence being observed in the four wild strains, intermediate in the two hybrid strains, and lowest in both domesticated strains (Table 1, Fig. 2). The incidence of MMP varied more within wild families than domesticated and hybrid families (Fig. 3). Among the wild strains, the incidence of MMP was highest in the Vosso strain (average 74.4%), and lowest in the Arna strain (average 42.6%). The lowest incidence of MMP was within Domestic 2 (average 4.8%).

The results for both approaches of splitting the fish into upper and lower size modes were very similar, and therefore, only results using the group-specific sizes are presented. The lower and upper size modes were split at 19 g in wild fish and at 26 g in hybrid and domesticated fish (Fig. 2). MMP were predominantly located in the lower size mode of all groups, where 280 out of 442 fish were MMP (63%), while 68 out of 486 fish were MMP in the upper mode (12%). Wild fish exhibited a more distinct bimodality and were more evenly distributed between the lower and upper size modes than hybrid and domesticated strains (Fig. 2).

**Fig. 4:**
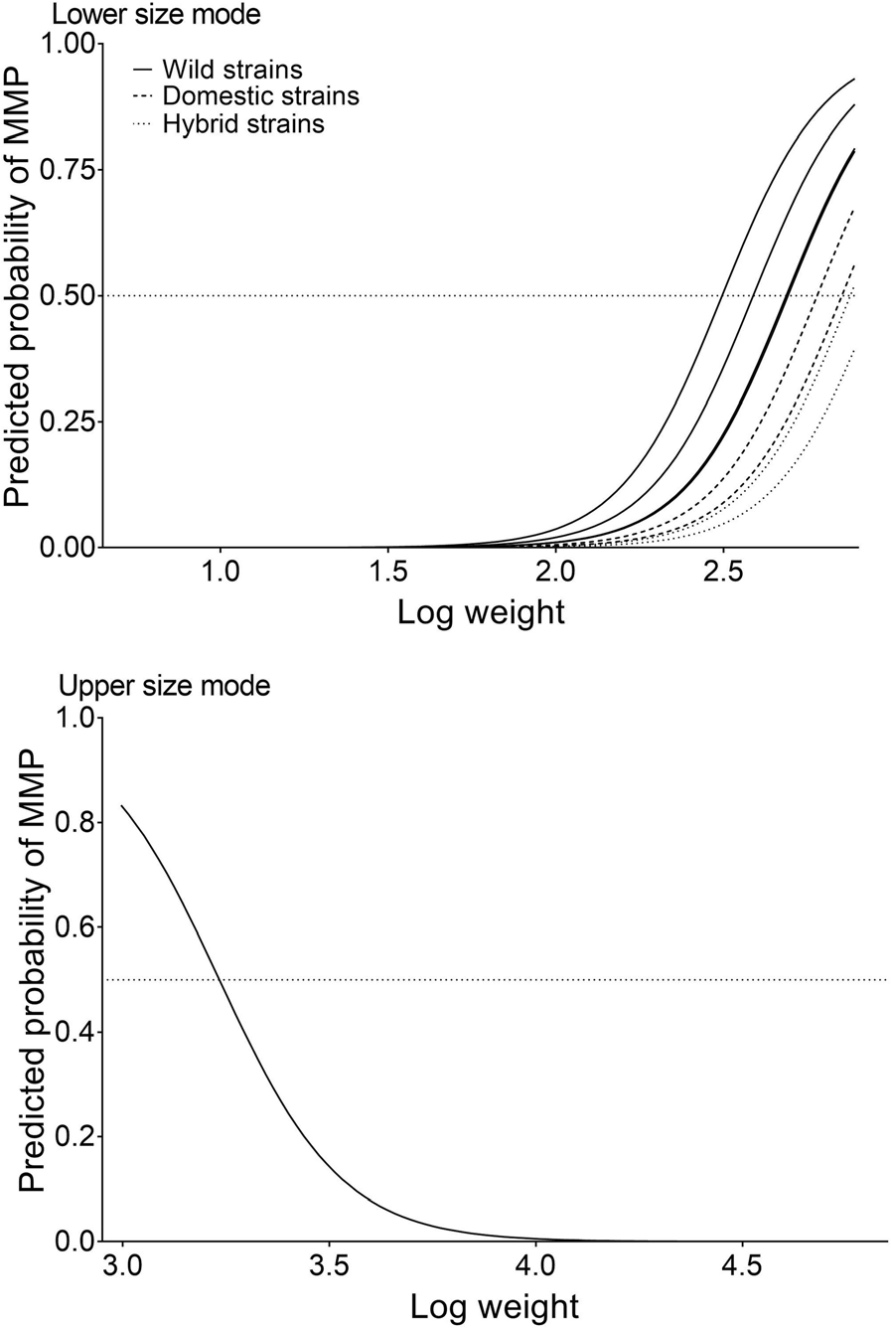
The predicted probability of maturing as a male parr over the individual logged weight range of the lower size mode for each strain and the upper size mode overall

The final model for the lower size mode contained strain, weight and the second-order polynomial of weight as fixed effects (Table 4A). Weight positively influenced the incidence of MMP and the second-order polynomial for weight had a negative influence on incidence for MMP, indicating that the incidence of MMP increased with size up to a maximum, after which the incidence of MMP decreased as size increased. After correction for multiple comparisons, there were no significant differences in the incidence of MMP between the domesticated strains and any of the wild or hybrid strains in the lower mode (Table 5). In the lower mode, the incidence of MMP in the hybrid strains were not significantly different to their domestic parental strains, but were significantly lower than their wild parental strains (Table 5). Furthermore, Hybrid 1 (44%) and Figgjo (55%) displayed the lowest percentage MMP, while Vosso (82%) and Arna (75%) displayed the highest percentage MMP. Driva displayed 62% MMP and Hybrid 2 displayed 58% MMP. Within the domesticated strains, Domestic 1 displayed 72% MMP and Domestic 2 displayed 67% MMP. The predicted probability of maturing as a male parr over the individual logged weight range of the lower size mode is presented in Fig. 4.

**Table 4:** Model selection for the fixed effects of the GLMM investigating incidence of mature male parr (MMP) in (A) the lower size mode and (B) the upper size mode

		Fixed				Random				
Model	Response	Strain	Weight	Weight^2	S x W	Dam	Sire	Tank	AIC	ΔAIC
A										
1	Sex	x	x	x	x	x	x	x	456.58	8.15
**2**		**x**	**x**	**x**		**x**	**x**	**x**	**448.43**	**0**
3		x	x			x	x	x	470.21	21.78
4		x		x		x	x	x	482.05	33.62
5			x	x		x	x	x	463.33	14.9
6		x				x	x	x	556.52	108.09
B										
1	Sex	x	x	x	x	x	x	x	229.71	10.39
2		x	x	x		x	x	x	227.14	7.82
3		x	x			x	x	x	225.15	5.83
4		x		x		x	x	x	225.53	6.21
5			x	x		x	x	x	221.28	1.96
6		x							321.27	101.95
**7**			**x**						**219.32**	**0**

AIC; Akaike information criterion. ΔAIC; difference in AIC value between the model and the final model. S x W; strain x average female family weight interaction. The final fixed effect structure is shown in bold

**Table 5:** *P* values of the Tukey adjusted multiple comparisons of the incidence of mature male parr (MMP) in the lower size mode between the strains. Significant p values are in bold

	Arna	Driva	Figgjo	Hybrid 1	Dom 1	Vosso	Hybrid 2	Dom 2
Arna	–							
Driva	0.96	–						
Figgjo	1.00	0.93	–					
Hybrid 1	0.07	**0.00**	**0.00**	–				
Dom 1	0.99	0.78	0.99	0.77	–			
Vosso	0.48	0.98	0.34	**0.00**	0.31	–		
Hybrid 2	0.58	0.09	0.42	0.99	0.99	**0.01**	–	
Dom 2	0.97	0.74	0.97	0.99	1.00	0.37	1.00	–

The final model for the upper size mode contained weight as a fixed effect (Table 4B). Weight was negatively associated with incidence of MMP, indicating that as size increased the incidence of MMP decreased (Table 5B). Strain was not significant, indicating that the incidence of MMP in the upper size mode did not differ significantly between any of the strains (Table 5B). The predicted probability of maturing as a male parr over the individual logged weight range of the upper size mode is presented in Fig. 4.

The random dam and sire effects were retained in all final models to account for family variation within strains, and tank was also retained in the final models.

## Discussion

This is the first study to simultaneously investigate growth and incidence of mature male parr (MMP) in multiple strains of domesticated, F1 wild/domesticated hybrid and wild Atlantic salmon reared under common aquaculture conditions. Upon termination of the experiment, domesticated salmon were between 2 and 5 times larger, and displayed between 3 and 19 times lower incidences of MMP than wild salmon. Hybrid strains displayed intermediate values for both traits overall. However, the incidence of MMP is highly influenced by growth (Fig. 2). When this was accounted for, by sorting fish into lower and upper size-modes, no difference in the incidence (%) of MMP were observed among domesticated and wild strains. Based upon these results, we find (1) no evidence that the size threshold for MMP has evolved in domesticated salmon, (2) the vastly lower incidence of MMP in domesticated strains, compared to wild strains under aquaculture conditions, is primarily due to their genetically increased growth rate causing them to outgrow the size threshold for early maturation, and (3) MMP incidence is highly likely to overlap among domesticated and wild salmon in the natural habitat where they tend to display overlapping growth rates, although hybrid offspring may display lower incidences of mature male parr.

### Growth of strains, sexes, and MMP

Domesticated salmon were larger in all comparisons, and in some cases, up to 5 times larger than the wild strains (Table 1, Fig. 2). Thus, even under a potentially growth-limiting 12-h light regime, domesticated salmon were still able to significantly outgrow wild salmon, and their hybrids tended to grow intermediate to both their parental strains. Therefore, growth of the hybrids was largely additive as has been observed in previous comparison studies [52–54]. Domesticated salmon grow faster and larger than wild salmon under farming conditions [31, 32, 53], but their growth differences are much less pronounced under semi-natural rearing conditions [53, 55], and in the wild [26, 33, 34].

The analysis of only the MMP found that most of the strains did not grow significantly different to each other after correction for multiple comparisons. Similarly, the growth analysis including MMP, and immature male and females, found that after correction for multiple comparisons, there was no significant difference in MMP weight between any of the strains. Any differences between the models (only the MMPs vs. all randomly sampled individuals) are probably due to the large growth differences between the MMP and the immature male and females coupled with the different corrections for the pairwise comparisons between the two models. Debes and Hutchings [35] also observed similar sizes of MMP between two domestic strains and their founder wild population, although MMP from their wild strain were on average slightly larger than the domestic strain, in contrast to the present study where there was a visible trend of hybrid and domesticated MMP being larger on average than wild MMP (Table 1, Fig. 2). These deviating results are probably due to different wild and domestic strains being used between the two studies, with different generations of selection in the domestic strains and different underlying genetics influencing growth.

In the present study, the strains were reared communally in a common garden design. Solberg et al. [53] compared the relative growth of domesticated, F1 hybrid and wild Atlantic salmon reared in single strain tanks and under similar common garden conditions and found no difference in the relative growth between the wild and domesticated strains across experimental designs. Therefore, it is unlikely that inter-strain competition influenced the growth differences observed in the present study.

### Relationship between growth and MMP incidence

The size-related trends for the incidence of MMP observed in the lower and upper size mode in the present study agree with the size threshold hypothesis of the alternative reproduction strategies of male Atlantic salmon. The significance of body size in the model for the lower size mode indicates that the incidence of MMP will increase as size increases towards the threshold for maturation (Fig. 4). Our results are in agreement with Piché et al. [14]*,* who also found that body size positively influenced the incidence of MMP in pure and hybrid crosses within and between three wild populations of Atlantic salmon. The significance of the second-order polynomial of body size in the model for the lower size mode indicates that the probability of maturation as a male parr increased as size increased towards the maturation threshold, and decreased as fish continued to grow past the maturation threshold into the upper size mode (Fig. 4). Therefore, within the lower size mode, fish had either attained the size threshold for maturation and thus matured as parr, or failed to reach the size threshold within the critical period and continued to grow and to potentially mature as parr at a later stage. Yates et al. [36] also demonstrated that the probability of maturation also exhibited a non-linear quadratic relationship with body size in one wild and two domesticated-wild hybrid Atlantic salmon strains at contrasting temperatures. Within the model for the upper size mode, the negative effect of weight indicates that the majority of the fish had exceeded the size threshold for maturation and continued to grow and potentially reach the smoltification threshold [18].

### Incidence of MMP among strains

In the present study, there was variation in the observed number of MMP among the strains; with wild strains displaying numerically more MMP than domesticated strains, and hybrid strains displaying intermediate numbers of MMP overall (Table 1, Fig. 2). Some strains which showed similar growth had very different overall levels of MMP (for example the Hybrid 1 strain vs. the Vosso strain, and Arna vs. Vosso Fig. 3). Similarly, wild strains with similar growth had visibly different levels of MMP (for example the Arna and Vosso strain, Fig. 3), demonstrating population level differences which are independent of size (i.e. a genetic component). Several studies have shown that threshold sizes linked to development of MMP may be population specific [14, 20, 56]. For example, Piché et al. [14] compared the incidence of MMP between pure and hybrid wild salmon populations and found that the predicted weight at 50% maturity differed between the crosses, with hybrids displaying intermediate maturation thresholds relative to the pure wild populations.

The model for the lower mode found that strain significantly influenced the incidence of MMP, however, after correction for multiple comparisons, there were no significant differences in the incidence of MMP between domesticated and wild strains. Although there were much fewer domesticated fish within the lower size mode, our results show that, when domesticated fish do grow in similar ranges as wild fish, the incidence of MMP is similar. Thus, it is likely that the overall low number of MMP observed in domesticated salmon in the present study is primarily the result of genetic changes in growth of domesticated salmon, causing them to outgrow the MMP threshold under farming conditions, and we found no evidence for an evolutionary change in the propensity to mature as a MMP, or the size threshold to mature as MMP. If the threshold for maturation had shifted in domesticated salmon, there would have been more domesticated MMP in the upper size mode (evolution of a higher size threshold) than wild MMP. In the model for the upper size mode, there was no difference between any of the strains for the incidence of MMP. Therefore, while the size threshold may not have evolved, the trait underlying the threshold (i.e. growth under aquaculture conditions) has evolved.

The incidence of MMP in the hybrid strains in the lower size mode was lower than their wild and domestic parental strains, although this was only statistically significant between hybrid and wild strains in the lower mode after correction for multiple comparisons. These results indicate a potential non-additive genetic effect causing a decrease in the incidence of MMP in the hybrid strains, although on average and across all sizes, the number of MMP in the hybrid strains were intermediate between their parental strains (Table 1, Figs. 3 and 4). Yates et al. [36] also reported that wild-domesticated hybrids displayed reduced levels of MMP compared to a wild strain. Similarly, lower levels of male parr maturity have been observed in backcrossed salmon that had undergone 3 and 5 generations of domestication relative to salmon from their wild ancestral strain [35]. In the wild, domesticated escapees often spawn with wild salmon and produce hybrids, and the observed lower incidence of MMP in hybrids in the present study could have implications for population fitness and maintenance of an important life-history trait in wild populations at risk of introgression from escaped domesticated salmon.

## Conclusions

The present study demonstrated that while strains of domesticated and hybrid salmon all displayed reduced numbers of MMP compared to several wild strains when reared together under farming conditions, this is primarily a response to selection for increased growth under these conditions (evolution of the underlying threshold trait), causing them to bypass the size threshold for parr maturation. Furthermore, we found no evidence that the size threshold for MMP has evolved in domesticated salmon, and our results indicate that the propensity to mature as parr is as likely in domesticated salmon as in wild salmon under natural conditions where their growth rates are observed to be similar [26, 33, 34].

Garant et al. [28] reported that MMP with a pure domesticated background had a higher reproductive and fertilisation success than domestic/wild hybrid and wild MMP, therefore, further genetic introgression via fish of domesticated background could be “fast-tracked” into wild populations through interbreeding with MMP ([28], but see [57]). Models designed to predict the influence of escaped domesticated salmon on wild populations found that including domesticated MMPs in the model increased the predicted decline of wild population genetic integrity contra to a model without MMP [29], indicating that this alternative life-history strategy may be an important vehicle for introgression. Although escaped domesticated salmon can be reproductively inferior to wild salmon under natural conditions [26, 27], genetic introgression from escaped domesticated salmon in native salmon populations is well-documented [25, 58, 59]. When salmon escape they often spawn with a wild salmon to produce hybrid offspring, and the lower incidence of MMP (% in the lower mode) observed in the hybrid strains in the present study indicates that the risk of further introgression via MMP offspring from domesticated parents may be lower if they produced hybrids than if they produce pure domesticated offspring in the wild. However, domesticated salmon may also escape as juveniles from hatcheries [60] or freshwater cages [61] directly into rivers supporting native populations. Introgression from juvenile escapes directly into rivers has been documented for example in Ireland [62]. If salmon escape into rivers prior to outgrowing the size threshold for maturation, the risk of introgression via domesticated MMP is likely, whereas if they escape into rivers after outgrowing the size threshold, the risk of introgression in this manner is reduced. Therefore, the risk of introgression via MMP will depend on whether the escaped salmon produce hybrid offspring with wild conspecifics or domesticated offspring with other escapees, and whether the escaped salmon themselves mature as parr, conditional on their size at the time of escapement.

While the risk of further introgression by MMP of hybrid offspring may be lower than for domesticated MMP offspring, the lower incidence of MMP observed in the hybrid strains reported here, and in Yates et al. [36] could indicate that introgression with domesticated salmon in general may decrease the maintenance of an important life history trait in wild populations. Therefore, the consequences of an escape event and any potential introgression resulting from it are complex. It is important that conservationists and river managers take these findings into account when managing wild salmon populations and estimating the risk of introgression from domesticated escapees. However, as our results and those of others have indicated variation in the body size threshold for maturation between wild populations [14], the relative incidence of MMP in offspring of domesticated salmon in the wild is likely to be river specific.

## Additional file

Additional file 1: Supplementary tables and figures. (DOCX 1405 kb)

Additional file 2: Dataset supporting the results. (XLSX 250 kb)
