# Supplementary material for: Implications for introgression: has selection for fast growth altered the size threshold for precocious male maturation in domesticated Atlantic salmon?
Source: BMC Evol Biol. 2018 Dec 18;18:188. doi: 10.1186/s12862-018-1294-y (PMC6298023; doi:10.1186/s12862-018-1294-y)
Supplement: Supplementary file 1 — Supplementary tables and figures. (DOCX 1405 kb) [file 12862_2018_1294_MOESM1_ESM.docx]

Additional File 1

Table S1: Experimental family crosses.

| \| Family \| Dam \| Sire \| Wild/domesticated \| Group \| \| --- \| --- \| --- \| --- \| --- \| \| 1 \| Wild A1 \| Wild A6 \| Wild \| Arna \| \| 2 \| Wild A2 \| Wild A7 \| Wild \| Arna \| \| 3 \| Wild A3 \| Wild A8 \| Wild \| Arna \| \| 5 \| Wild A4 \| Wild A9 \| Wild \| Arna \| \| 6 \| Wild A5 \| Wild A10 \| Wild \| Arna \| \| 13 \| Wild F1 \| Wild F6 \| Wild \| Figgjo \| \| 14 \| Wild F1 \| Dom D11 \| Hybrid 1 \| Figgjo x Dom 1 \| \| 15 \| Wild F2 \| Wild F7 \| Wild \| Figgjo \| \| 16 \| Wild F2 \| Dom D6 \| Hybrid 1 \| Figgjo x Dom 1 \| \| 17 \| Wild F3 \| Wild F8 \| Wild \| Figgjo \| \| 18 \| Wild F3 \| Dom D7 \| Hybrid 1 \| Figgjo x Dom 1 \| \| 19 \| Wild F4 \| Wild F9 \| Wild \| Figgjo \| \| 20 \| Wild F4 \| Dom D12 \| Hybrid 1 \| Figgjo x Dom 1 \| \| 25 \| Wild F5 \| Wild F10 \| Wild \| Figgjo \| \| 26 \| Wild F4 \| Dom D9 \| Hybrid 1 \| Figgjo x Dom 1 \| \| 34 \| Dom 1 D1 \| Dom 1 D6 \| Dom \| Dom 1 \| \| 36 \| Dom 1 D2 \| Dom 1 D7 \| Dom \| Dom 1 \| \| 40 \| Dom 1 D3 \| Dom 1 D8 \| Dom \| Dom 1 \| \| 44 \| Dom 1 D4 \| Dom 1 D9 \| Dom \| Dom 1 \| \| 48 \| Dom 1 D5 \| Dom 1 D10 \| Dom \| Dom 1 \| \| 49 \| Dom 2 DD1 \| Dom 2 DD6 \| Dom \| Dom 2 \| \| 50 \| Dom 2 DD1 \| Wild V11 \| Hybrid 2 \| Dom 2 x Vosso \| \| 51 \| Dom 2 DD2 \| Dom 2 DD7 \| Dom \| Dom 2 \| \| 52 \| Dom 2 DD2 \| Wild V6 \| Hybrid 2 \| Dom 2 x Vosso \| \| 53 \| Dom 2 DD3 \| Dom 2 DD8 \| Dom \| Dom 2 \| \| 54 \| Dom 2 DD3 \| Wild V7 \| Hybrid 2 \| Dom 2 x Vosso \| \| 57 \| Dom 2 DD4 \| Dom 2 DD9 \| Dom \| Dom 2 \| \| 58 \| Dom 2 DD4 \| Wild V9 \| Hybrid 2 \| Dom 2 x Vosso \| \| 63 \| Dom 2 DD5 \| Dom 2 DD10 \| Dom \| Dom 2 \| \| 64 \| Dom 2 DD5 \| Wild V10 \| Hybrid 2 \| Dom 2 x Vosso \| \| 66 \| Wild V1 \| Wild V6 \| Wild \| Vosso \| \| 67 \| Wild V2 \| Wild V7 \| Wild \| Vosso \| \| 68 \| Wild V3 \| Wild V8 \| Wild \| Vosso \| \| 69 \| Wild V4 \| Wild V9 \| Wild \| Vosso \| \| 72 \| Wild V5 \| Wild V10 \| Wild \| Vosso \| \| 73 \| Wild Dr1 \| Wild Dr4 \| Wild \| Driva \| \| 75 \| Wild Dr2 \| Wild Dr5 \| Wild \| Driva \| \| 76 \| Wild Dr2 \| Wild Dr4 \| Wild \| Driva \| \| 78 \| Wild Dr3 \| Wild Dr5 \| Wild \| Driva \| |  |  |  |  |  |  |
| --- | --- | --- | --- | --- | --- | --- | --- | --- | --- | --- | --- | --- | --- | --- | --- | --- | --- | --- | --- | --- | --- | --- | --- | --- | --- | --- | --- | --- | --- | --- | --- | --- | --- | --- | --- | --- | --- | --- | --- | --- | --- | --- | --- | --- | --- | --- | --- | --- | --- | --- | --- | --- | --- | --- | --- | --- | --- | --- | --- | --- | --- | --- | --- | --- | --- | --- | --- | --- | --- | --- | --- | --- | --- | --- | --- | --- | --- | --- | --- | --- | --- | --- | --- | --- | --- | --- | --- | --- | --- | --- | --- | --- | --- | --- | --- | --- | --- | --- | --- | --- | --- | --- | --- | --- | --- | --- | --- | --- | --- | --- | --- | --- | --- | --- | --- | --- | --- | --- | --- | --- | --- | --- | --- | --- | --- | --- | --- | --- | --- | --- | --- | --- | --- | --- | --- | --- | --- | --- | --- | --- | --- | --- | --- | --- | --- | --- | --- | --- | --- | --- | --- | --- | --- | --- | --- | --- | --- | --- | --- | --- | --- | --- | --- | --- | --- | --- | --- | --- | --- | --- | --- | --- | --- | --- | --- | --- | --- | --- | --- | --- | --- | --- | --- | --- | --- | --- | --- | --- | --- | --- | --- | --- | --- | --- | --- | --- | --- | --- | --- | --- | --- | --- | --- | --- | --- | --- |
|  |  |  |  |  |  |  |

Table S2: Output of the final model for the LME investigating growth among strains and maturation status.

|  | N | Response | Random | effects |  |  |  | Fixed | effects |  |  |  |  |  |  |
| --- | --- | --- | --- | --- | --- | --- | --- | --- | --- | --- | --- | --- | --- | --- | --- |
|  |  | Variable | Variable | Chi.sq | Chi.df | P value |  | Variable | Sum Sq | Mean Sq | Num Df | Den Df | F value |  | P value |
|  | 1988 | Log Weight | **Tank** | **4.24** | **1** | **0.0395** |  | **Strain x Sex** | **41.64** | **2.97** | **14** | **1937.33** | **8.25** |  | **<1e-07** |
|  |  |  | **Sire** | **48.88** | **1** | **<1e-07** |  | **Strain** | **31.77** | **4.54** | **7** | **26.47** | **12.58** |  | **0** |
|  |  |  | **Dam** | **75.27** | **1** | **<1e-07** |  | **Sex** | **60.59** | **30.29** | **2** | **1937.33** | **84.00** |  | **<1e-07** |

N; number of fish. Log weight; log10 (wet weight + 1) at termination. Chi.sq; the value of the Chi square statistics. Chi Df; the degrees of freedom for the test. P value; P-value of the likelihood ratio test for the random effect. Sum.Sq; sum of squares. Num Df, numerator degrees

of freedom. Den Df; denominator degrees of freedom based on Sattherwaithe’s approximations. F; F-value. The variables in bold were retained in the final model.

Table S3: P values of the Tukey adjusted multiple comparisons of weight among the three sex categories; (A) mature male parr (MMP); (B) immature male parr; and (C) females. Significant comparisons are shown in bold. Dom; Domesticated.

| A | Arna | Driva | Figgjo | Hybrid 1 | Dom 1 | Vosso | Dom 2 | Dom 2 |
| --- | --- | --- | --- | --- | --- | --- | --- | --- |
| Arna | - |  |  |  |  |  |  |  |
| Driva | 0.99 | - |  |  |  |  |  |  |
| Figgjo | 0.98 | 1.00 | - |  |  |  |  |  |
| Hybrid 1 | 1.00 | 0.60 | 0.05 | - |  |  |  |  |
| Dom 1 | 0.97 | 0.27 | 0.14 | 1.00 | - |  |  |  |
| Vosso | 1.00 | 1.00 | 1.00 | 0.99 | 0.81 | - |  |  |
| Hybrid 2 | 1.00 | 0.67 | 0.50 | 1.00 | 1.00 | 0.97 | - |  |
| Dom 2 | 1.00 | 0.90 | 0.88 | 1.00 | 1.00 | 1.00 | 1.00 | - |
| B | Arna | Driva | Figgjo | Hybrid 1 | Dom 1 | Vosso | Hybrid 2 | Dom 2 |
| Arna | - |  |  |  |  |  |  |  |
| Driva | **0.03** | - |  |  |  |  |  |  |
| Figgjo | **0.00** | 1.00 | - |  |  |  |  |  |
| Hybrid 1 | 1.00 | **0.04** | **0.00** | - |  |  |  |  |
| Dom 1 | 0.33 | **0.00** | **0.00** | 0.11 | - |  |  |  |
| Vosso | 0.75 | 0.92 | 0.76 | 0.84 | **0.00** | - |  |  |
| Hybrid 2 | 1.00 | **0.00** | **0.00** | 0.99 | 0.96 | **0.04** | - |  |
| Dom 2 | 0.24 | **0.00** | **0.00** | 0.14 | 1.00 | **0.00** | 0.36 | - |
| C | Arna | Driva | Figgjo | Hybrid 1 | Dom 1 | Vosso | Hybrid 2 | Dom 2 |
| Arna | - |  |  |  |  |  |  |  |
| Driva | 0.54 | - |  |  |  |  |  |  |
| Figgjo | 0.01 | 1.00 | - |  |  |  |  |  |
| Hybrid 1 | 1.00 | 0.46 | 0.00 | - |  |  |  |  |
| Dom 1 | 0.17 | 0.00 | 0.00 | 0.10 | - |  |  |  |
| Vosso | 1.00 | 0.58 | 0.01 | 1.00 | 0.14 | - |  |  |
| Hybrid 2 | 1.00 | 0.19 | 0.00 | 1.00 | 0.58 | 1.00 | - |  |
| Dom 2 | 0.23 | 0.00 | 0.00 | 0.28 | 1.00 | 0.19 | 0.14 | - |


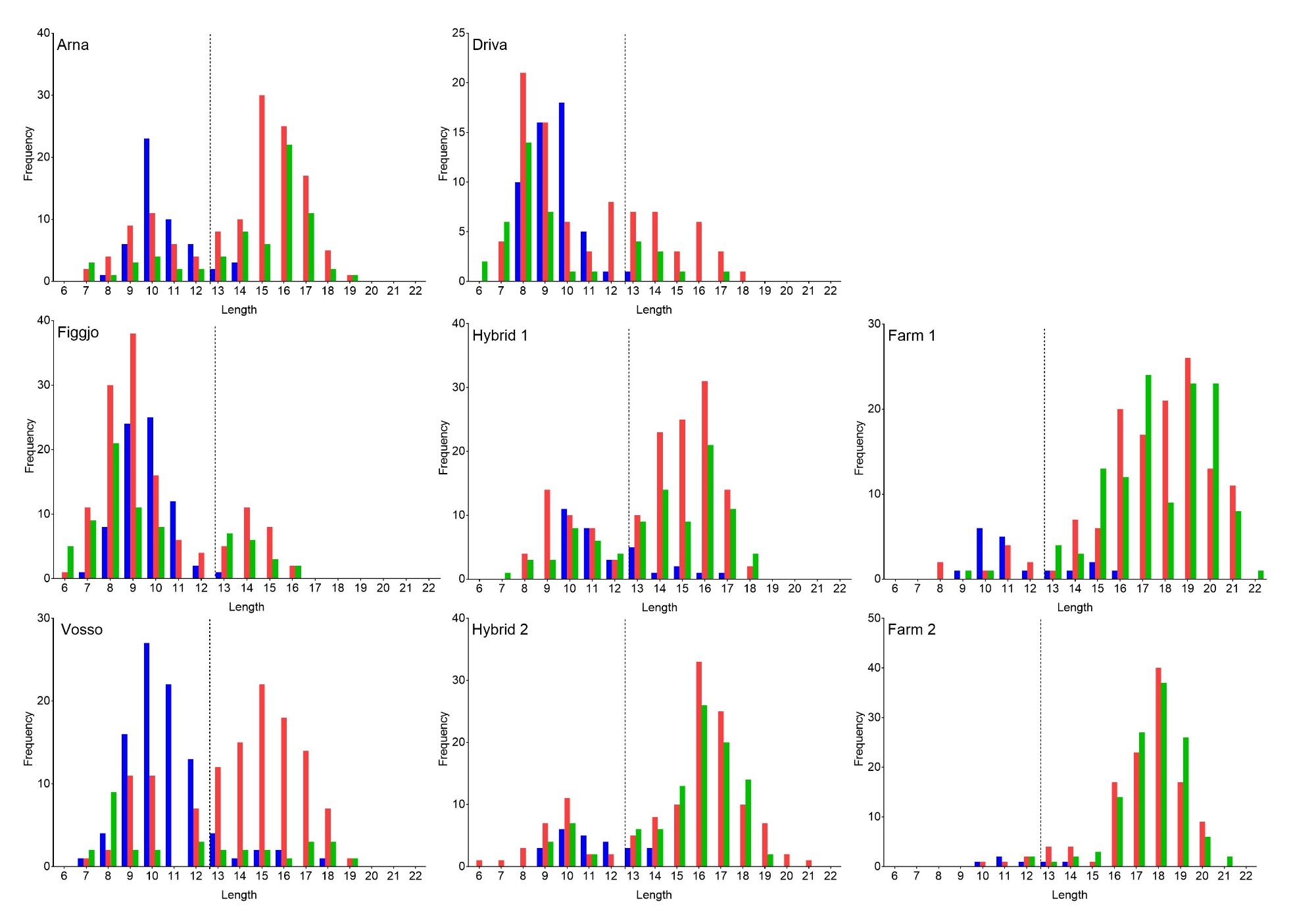


Figure S1: Length frequency distributions for each strain. The horizontal stippled line indicates the chosen threshold between the lower and upper mode for these data combined (13cm). Note scaling differences on Y-axis


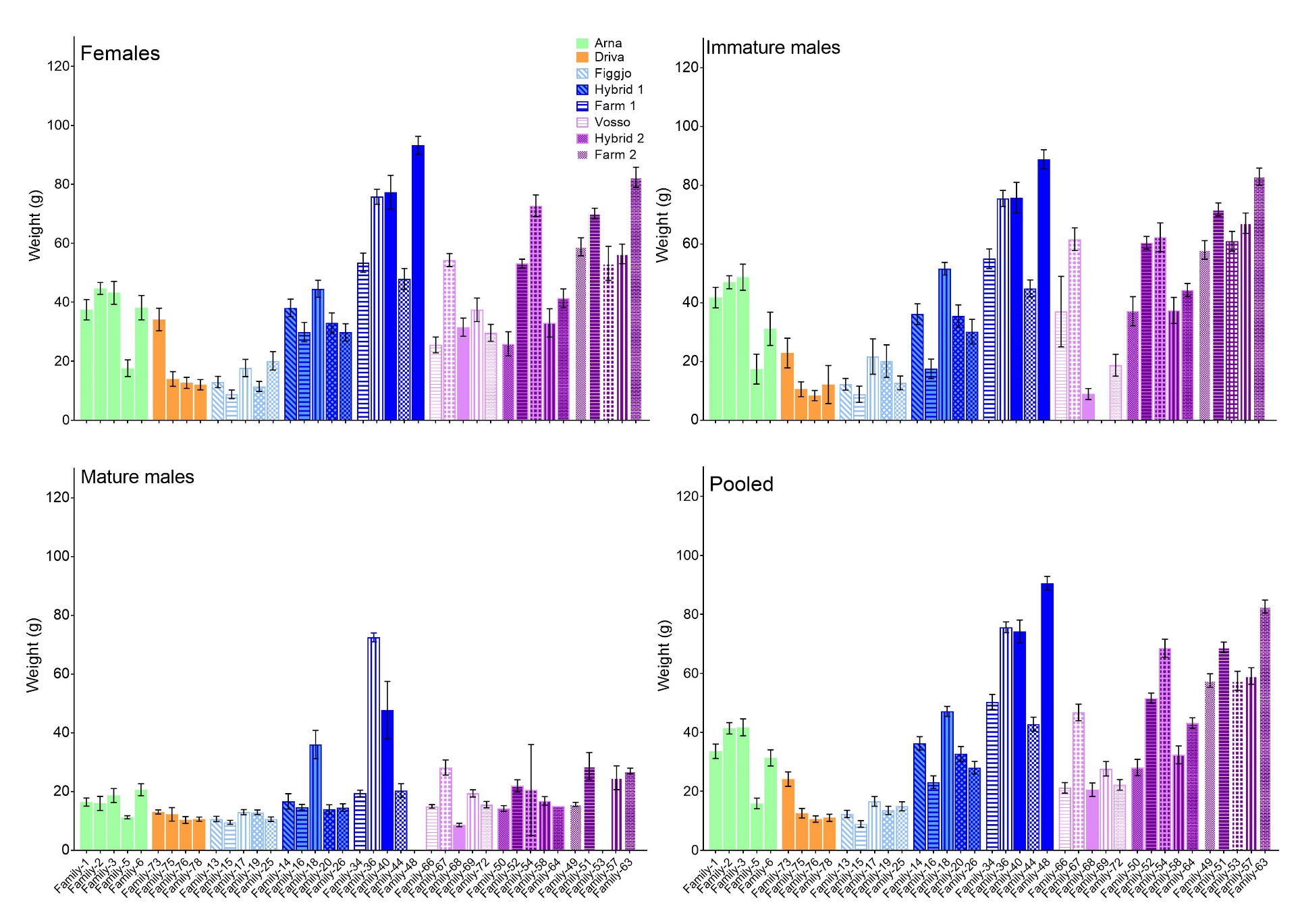


Figure S2: Average family weight per strain for each of the three sex categories: Females (top left), immature males (top right) and mature males (bottom left) and for the pooled representative sampling (bottom right). The average family weight of the mature male parr was calculated using all available MMP data. Half siblings are illustrated using patterns and colours.


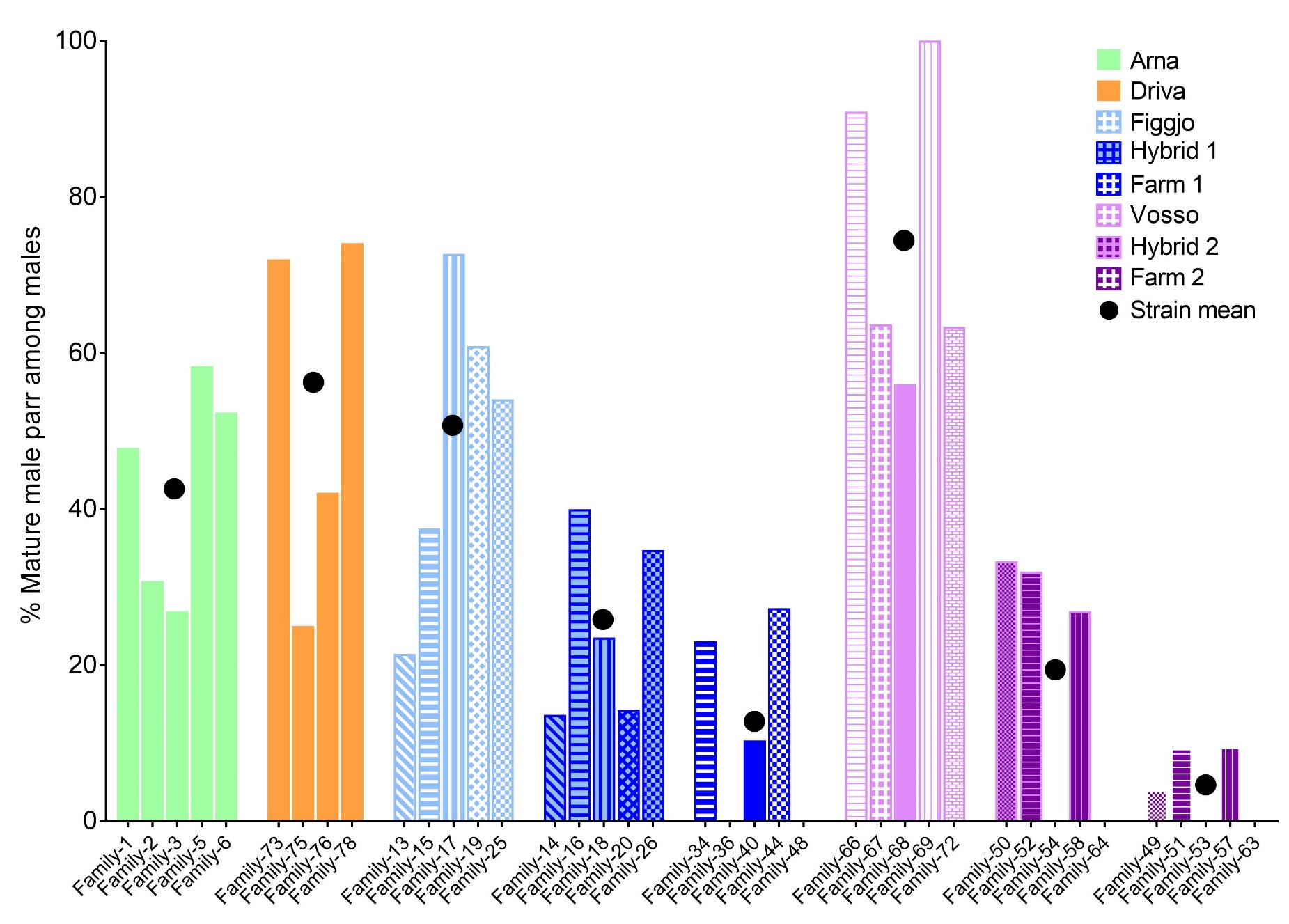


Figure S3: Percentage mature male parr (MMP) among the males in each family. The strain mean is displayed by the black dots.

Hybrids and their domesticated or wild half siblings are illustrated by matching patterns and colours.
